# Cemented Bipolar Hemiarthroplasty as a Treatment Modality for Pathological Fracture in the Proximal Femur Metastasis Without the Recurrence of Primary Breast Cancer: A Case Report

**DOI:** 10.7759/cureus.64326

**Published:** 2024-07-11

**Authors:** Tushar Chaudhari, Mukesh O Phalak, Ajinkya K Chaudhari

**Affiliations:** 1 Department of Orthopaedics, Dr D.Y. Patil Medical College, Hospital and Research Institute, Pune, IND

**Keywords:** cemented bipolar prosthesis, pathological femur fracture, solitary bone metastasis, bipolar hemi-arthroplasty, skeletal metastases

## Abstract

Metastatic lesions in the proximal femur are well-known in the literature and are important since they can progress to pathological fractures and impair the patient's mobility. We present the case of a middle-aged female with a history of breast carcinoma 20 years ago, who experienced diffuse chronic hip pain for the past two months. Radiographs, MRI, and PET scans revealed a metastatic lesion in her proximal femur. After consulting with an oncologist, it was determined that adjuvant chemoradiotherapy was unnecessary. The treatment strategy was dependent on the preoperative general health condition, the life expectancy, amount of metastasis, bone quality, pathological fractures and factors affecting the union and capacity to ambulate the patient postoperatively. The patient underwent a cemented bipolar hemiarthroplasty to excise all metastatic tissue and provide a painless, functional, and mobile joint. Bipolar hemiarthroplasties articulate at two levels, and this dual-bearing design is believed to reduce acetabular wear. The bipolar hemiarthroplasty also eliminated the risk of complications associated with the acetabular component, which would necessitate early revision surgery. Modular bipolar hemiarthroplasty is a good modality of replacement associated with fewer complications and improves quality of life.

## Introduction

Bone is a common site for metastasis, mainly in the spine, pelvis, ribs, skull, and femur, leading to pain and pathologic fractures [1]. These metastases arise from primary lesions from the breast, kidney, leukemias, and thyroid and are at high risk of pathological fracture [2,3]. In a metastatic lesion, the proximal femur, i.e. the neck of the femur, intertrochanteric region and the subtrochanteric regions due to mechanical factors like weight bearing and vascularity of metastasis, which adds to mechanical instability and increased calcium resorption are prone to the pathological fractures. The majority of the lesions in these areas with pain are pathologic fractures, which lead to disability and restricted ambulation [2]. The main objective of treatment in such individuals is increasing life expectancy, improving quality of life, and rehabilitation to maintain function for basic activities of daily living. Hence apt treatment modality is very important for good prognosis but it varies from case to case so a standard management of choice is difficult to establish depending on the surgeon’s choice. Modalities of treatment include fixation with intramedullary nailing systems, dynamic hip screw, bipolar hemiarthroplasty, and total hip arthroplasty with additional options like megaprosthesis and calcar replacing versions. Reconstruction is one such important modality, which offers excision of the lesion, pain control, stability of the joint, restoration of function and immediate weight bearing [4-6]. We present a case of proximal femur fracture metastasis from most probably breast primary managed with cemented bipolar hemiarthroplasty.

## Case presentation

A 53-year-old female presented to us with pain in the right hip pain for a duration of 2 months. The pain was insidious in onset, progressive in nature, and dull aching type of pain aggravated on walking and exertion. The onset of pain was not associated with any fall or injury to the hip. The patient did not give any complaints of such a complaint in the past or any association with other joint pain or back pain. The patient gave a history of breast cancer in the right breast 20 years ago for which a right mastectomy was done along with preoperative and postoperative chemotherapy and radiation 20 years ago, details of which were not available to the patient. Since then, the patient has not had any symptoms or lesions diagnosed as metastasis or recurrence of breast carcinoma and was asymptomatic. On clinical evaluation, the patient had difficulty in walking due to pain and she preferred ambulation in a wheelchair. There were no associated deformities of the hip joint in a squared pelvis and no limb length discrepancy. There was no local rise in temperature or superficial tenderness. The patient had a deep tenderness on the right hip. The range of motion of the right hip was restricted and was painful terminally. The patient had a restricted range of motion of the right hip joint with the inability to actively flex the hip and the Active Straight Leg Raise (SLR) test was positive. The patient did not have any abductor weakness, hip impingement, or rotational malalignment of lower limbs with a normal spine and sacroiliac joint on clinical examination.

**Figure 1:**
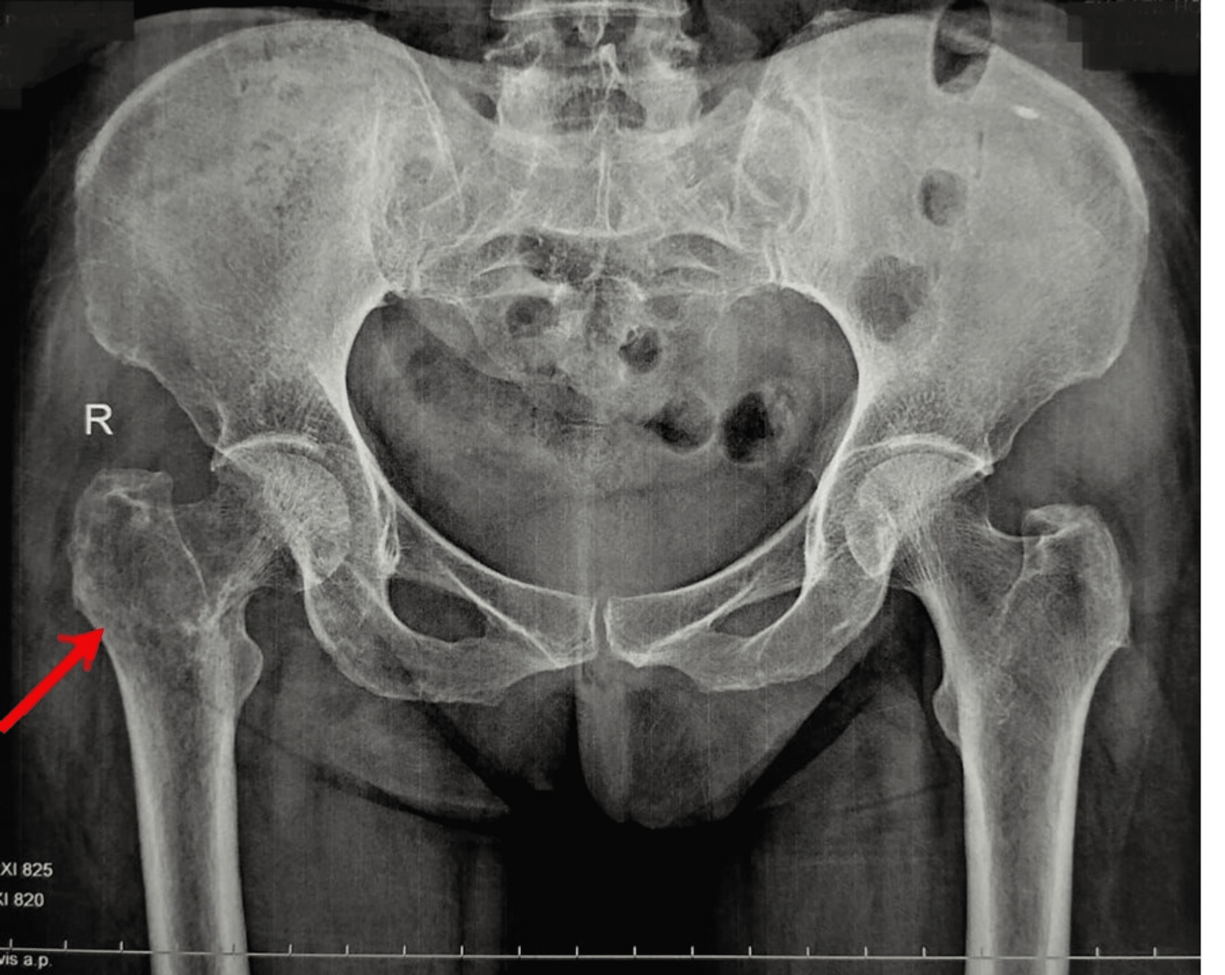
Preoperative X-ray pelvis with both hips showing radioluscent lesion with discontinuity of the cortex of calcar indicating a pathological fracture in the right proximal femur (arrow) The red arrow mark shows a discontinuity in the calcar of the right side indicating a pathological fracture with coxa vara on the right side when compared with the left side of the femur.

**Figure 2:**
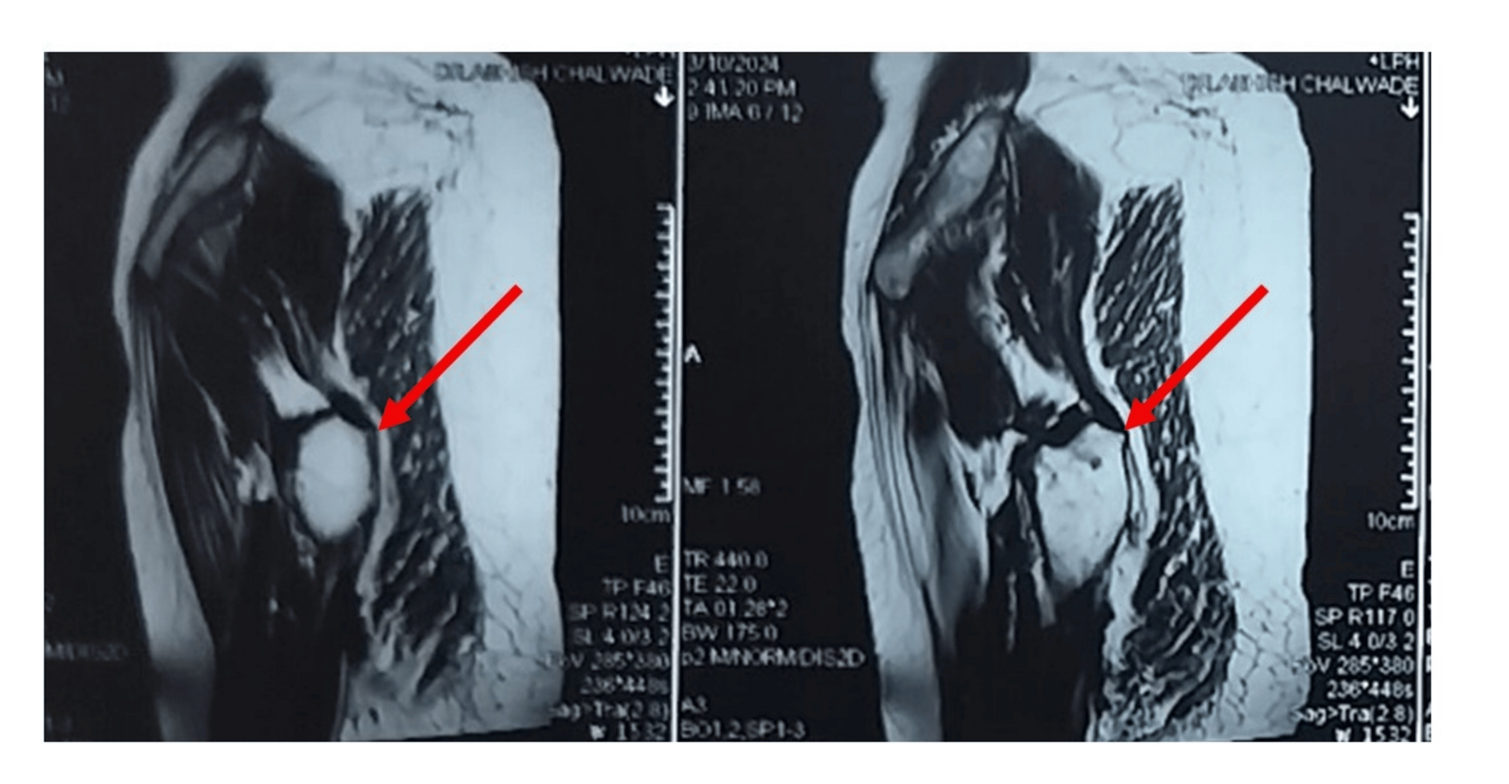
Sagittal MRI pelvis with both hips cuts showing proximal femoral metastasis The red arrow marks shows a lesion in the intertrochanteric area contained in the bony cavity but significantly occupying the proximal femur.

**Figure 3:**
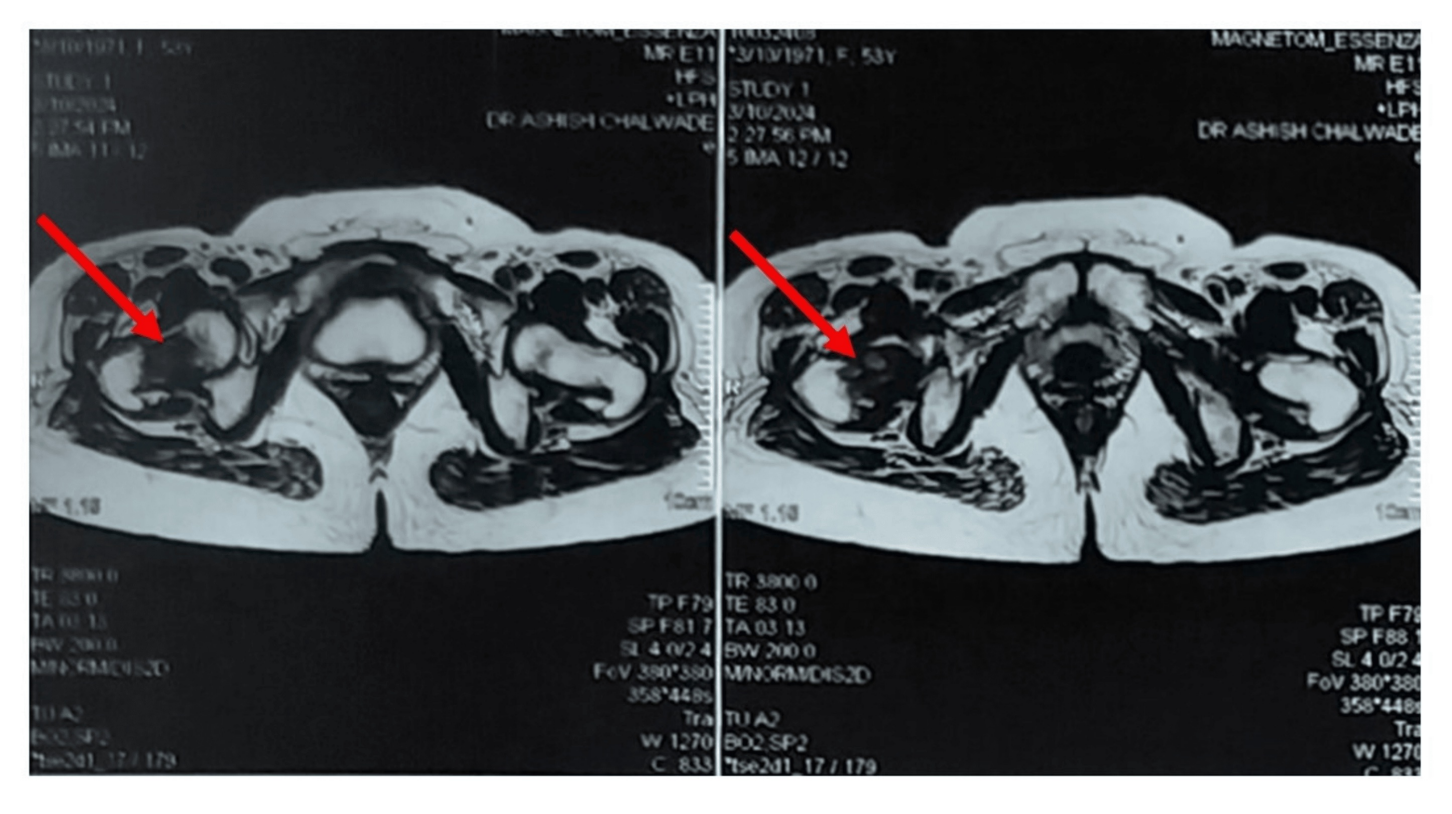
Axial MRI pelvis with both hips cuts showing proximal femoral metastasis The red arrow marks show a hypointense shadow in the axial cuts of the MRI, indicating the involvement of the neck of the femur and the intertrochanteric area.

**Figure 4:**
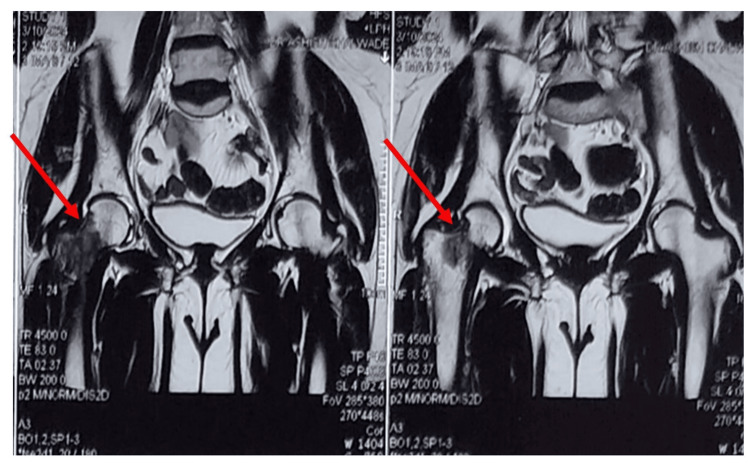
Coronal MRI pelvis with both hips cuts showing proximal femoral metastasis The red arrow marks show a hypointense lesion in the neck and intertrochanteric area.

**Figure 5:**
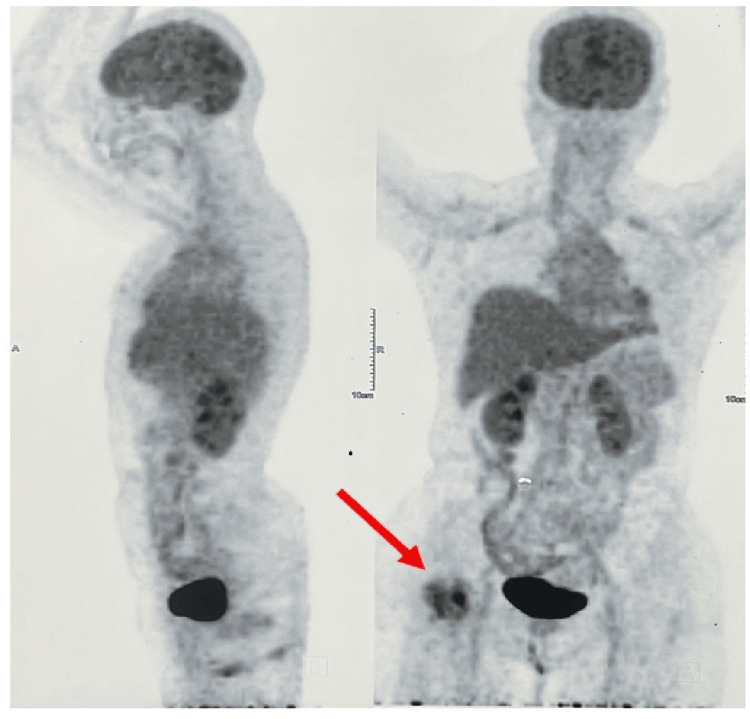
18-fluorodeoxyglucose (FDG) PET CT scan The red arrow mark shows tracer uptake with mixed density in the proximal femur area indicative of a neoplastic etiology.

The patient had been investigated by an X-ray (Figure 1) suggestive of an osteolytic intramedullary lesion. An MRI pelvis with both hips (Figures 2, 3, 4) was done at the onset of pain roughly 1 month before presenting to the orthopaedic surgeon which suggested a fibrous lesion in the intertrochanteric region with no pathological fracture. On clinical suspicion of a pathological fracture and a history of breast carcinoma an F-18 fluorodeoxyglucose (FDG) whole-body PET CT scan was done. The PET CT (Figure 5) demonstrated a moderate uptake of the tracer in a mixed-density lesion in the right femoral neck extending into the head trochanteric region and proximal shaft with a cortical break suggestive of a pathological fracture, most probably a metastasis, considering the history. There was no recurrence seen in the breast.

**Figure 6:**
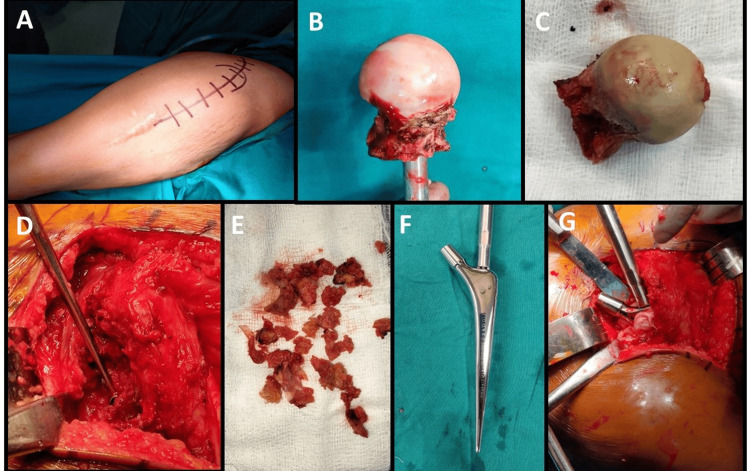
Intraoperative images The intraoperative findings showing: A – Posterior approach to the hip, B and C – Extracted head with neck, D – Metastatic bone removed with curettage, E – Extracted metastatic bone, F – Bipolar hemiarthroplasty cemented stem design, G – Implanted cemented stem

**Figure 7:**
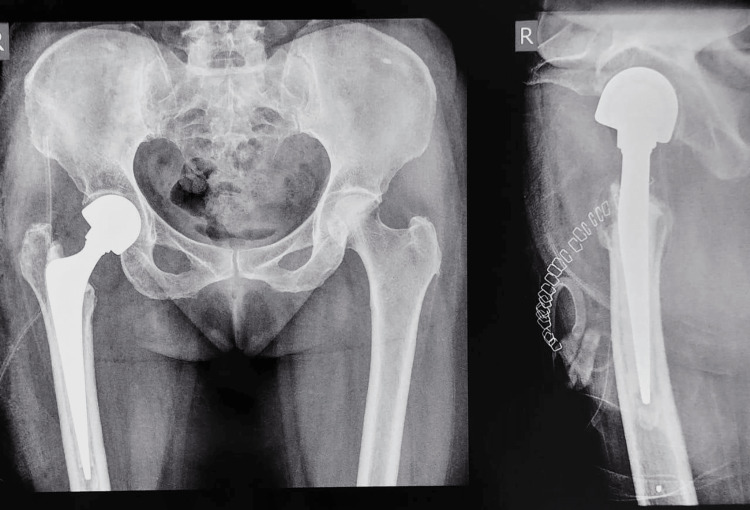
Postoperative X-ray - Right-sided cemented bipolar hemiarthroplasty The X-ray shows the patient is operated on with a right-sided bipolar hemiarthroplasty; the radiolucent shadow of metastatic lesion in the preoperative X-ray is absent and has been replaced with the bipolar hemiarthroplasty prosthesis; the prosthesis has an intramedullary radioopaque shadow surrounding the stem of the bone cement; and in the lateral X-ray, a radioopaque dot can be seen of the cement restricter.

We evaluated the treatment modalities and compared between proximal femoral resection with a megaprosthesis replacement as against a cemented bipolar hemiarthroplasty and found that bipolar hemiarthroplasty offers benefits like shorter procedure with lesser blood loss, lesser hardware intensive, lesser complications, considering finite life of implant and periprosthetic complications of replacement surgeries easier, subsequent surgeries which can provide better functional outcome, and hence, decided to opt for reconstruction, since it would remove the localised intramedullary metastasis region and replace it with a functional hip. The oncosurgeon suggested that there was no role of preoperative role of chemoradiotherapy and that we could proceed with our plan. In modalities of reconstruction bipolar hemiarthroplasty serves as a better modality since complications of acetabular component in total hip arthroplasty with subsequent difficulties in revision surgeries outweighed the benefits of total of hip replacement. The patient was operated with cemented bipolar hemiarthroplasty of the hip (Figures 6, 7) with a view of achieving stability and to tackle the residual metastasis with cementation with a view of local exothermic reaction of cementation reducing the risk of recurrence of metastasis. Intraoperatively, a pathological fracture was present in the basicervical neck of the femur. We excised the head; the neck and proximal femur lesion was excised in the box cut, which we took deeper than usual to remove as much metastatic bone tissue as possible, and further, the lytic part was extracted using a bone nibbler and surgical scoop (Figure 6). This excised tissue was sent for histopathological examination. Proximal femoral reaming was done for ensuring complete removal of metastatic tissue and 5 liters of normal saline sterile wash was given followed by standard procedure of cemented bipolar hemiarthroplasty.

**Figure 8:**
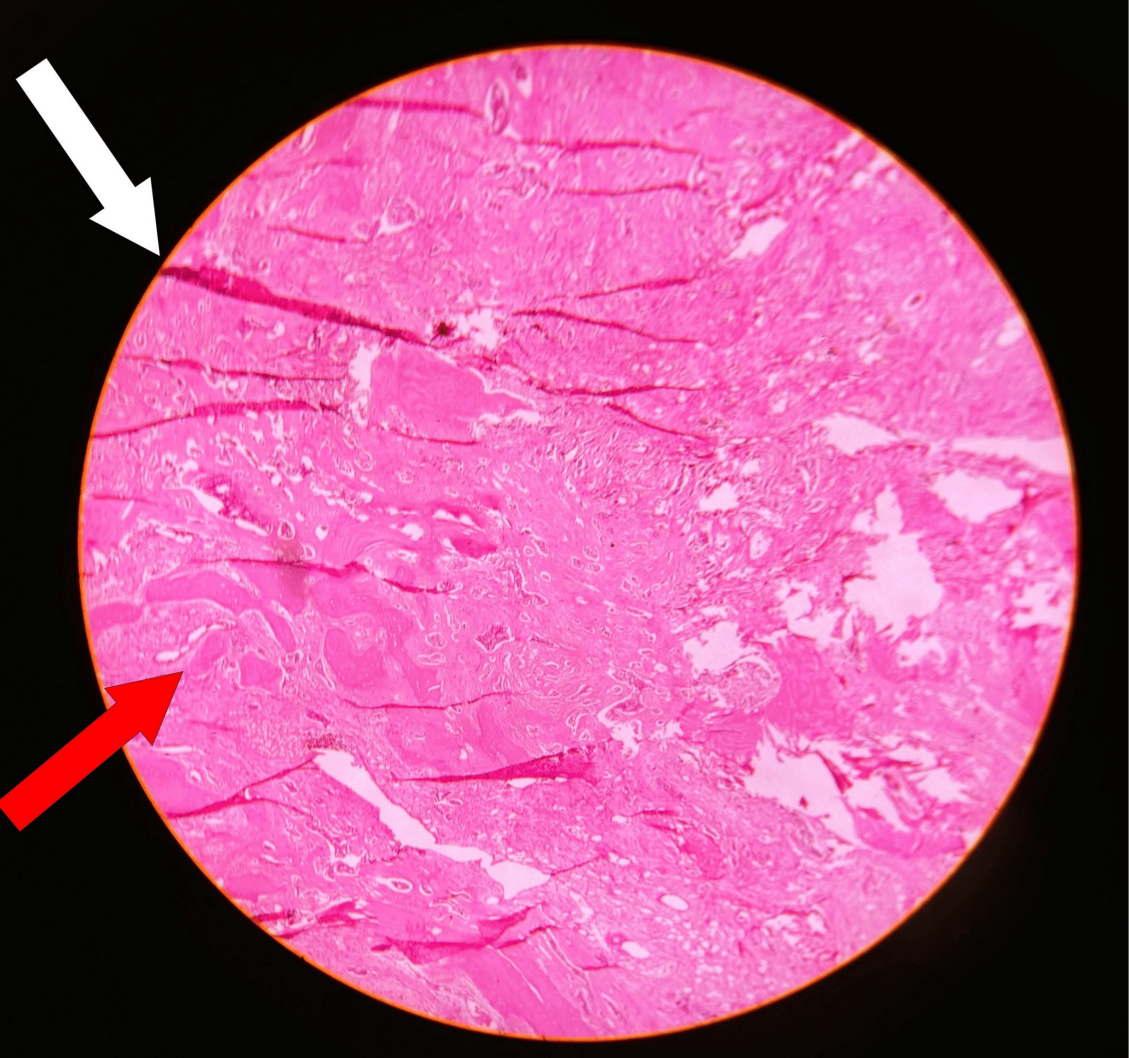
Histopathological examination of the excised specimen The histopathological examination showing areas of hemorrhage marked with the white arrow and the red arrow showing cells arranged in nests and cords with large hyperchromatic pleomorphic nuclei and eosinophilic cytoplasm

The histopathological examination (Figure 8) showed bony tissue infiltrated with tumor cells which were large hyperchromatic pleomorphic nuclei and eosinophilic cytoplasm with areas of hemorrhage and necrosis. A proper, staged physiotherapy protocol was used for rehabilitation. The patient was ambulated immediately the next day with a walker that was used by the patient for 2 weeks, following which the patient became full weight bearing with assistance of a walker or a walking stick. In 2 weeks, the patient was back to her normal life without any restriction in her activities of daily living with a stable, functional, painless hip.

## Discussion

The proximal femur is often affected by bone metastases, leading to frequent occurrences of pathologic fractures due to the region's biomechanical properties and the delayed identification of metastases [2,7]. There is approximately a risk of 8% - 30% of pathological fractures in metastatic lesions at the time of presentation and it is considered an important prognostic factor [8-10]. Surgical interventions in these cases are primarily palliative, aiming to alleviate pain and support weight-bearing. Modular prostheses have been shown to extend survival following aggressive treatment of bone metastases [1,11]. Furthermore, evidence indicates that resecting a solitary metastasis can improve prognosis in patients with favorable tumor types, including those with breast, prostate, kidney, bowel, thyroid cancers, or myeloma [11-13]. We demonstrated the oncologic and functional outcome of a patient undergoing replacement surgery for a pathological fracture in the proximal femoral region with cemented bipolar hemiarthroplasty.

Capanna and Campanacci proposed a treatment algorithm in 2001 for metastases in long bones and the pelvis, categorizing patients into four classes: 1) solitary lesions 2) pathologic fractures 3) impending fractures and 4) other lesions [14,15]. Factors such as loss of mobility, functional independence, bed rest, deep vein thrombosis risk, the necessity of surgical intervention, and the primary cancer's biological behavior are considered potential causes [6]. The current surgical goal is to relieve pain and ensure a painless, mobile, stable joint with early full-weight mobilization. Life expectancy, assessed through a multidisciplinary approach, is a crucial factor in selecting surgical treatment. It is essential that the survival of the implant surpasses the overall survival of the patient to avoid complex and potentially fatal reoperations, which increase morbidity and health decline [16,17].

Local adjuvant treatments have been used to enhance intralesional surgical outcomes, including various radiation therapy protocols, concurrent tumor debulking, and bone cement augmentation [18]. Limited complications and good functional outcomes have been observed with bipolar prostheses in cases of proximal femoral metastasis contained in the proximal area of femur with no spread to adjacent tissue of acetabular involvement [19]. In our case, radiotherapy was not required due to the absence of other metastatic lesions and the resection of the proximal femur. However, radiotherapy is associated with increased risks of deep infection and thromboembolic complications, as it renders tissues fragile, necrotic, and chronically ischemic [20]. Literature also suggests that it is advisable to use cemented stems for reconstruction in patients with bone metastasis in the proximal femoral region to reduce complications and for a favorable outcome [14].

## Conclusions

The proximal femur is one of the common sites of metastasis and the best modality of treatment is debatable and depends on patient comorbidities, the severity of metastasis and life expectancy which play a key role in surgeons’ choice of management. The cemented bipolar hemiarthroplasty serves as a viable means of reconstruction in proximal femoral metastasis. Bipolar hemiarthroplasty is a good treatment modality in such patients with a pathological fracture having a good implant life with a possible conversion of total hip arthroplasty or revision as required later. It provides a good functional outcome in terms of range of motion and early full weight-bearing ambulation and is associated with a low complication rate and good stability of the hip joint. However, each case is unique and all factors have to be considered to decide the best possible plan after assessing the risk-benefit ratio, and a multidisciplinary approach should be followed to have a holistic approach to decide the final plan of management giving a painless, stable, and a good functional outcome of the surgery.
